# Isolongifolene-loaded chitosan nanoparticles synthesis and characterization for cancer treatment

**DOI:** 10.1038/s41598-022-23386-4

**Published:** 2022-11-10

**Authors:** Dharmar Manimaran, Namasivayam Elangovan, Panagal Mani, Kumaran Subramanian, Daoud Ali, Saud Alarifi, Chella Perumal Palanisamy, Hongxia Zhang, Kowsalya Rangasamy, Vasan Palanisamy, Renuka Mani, Kavitha Govarthanan, Wilson Aruni, Rajeshkumar Shanmugam, Guru Prasad Srinivasan, Aruncahllam Kalirajan

**Affiliations:** 1 Department of Biotechnology, School of Biosciences, Periyar University, Salem, Tamil Nadu 636011 India; 2 Department of Animal Nutrition, Veterinary College and Research Institute, Tamil Nadu Veterinary and Animal Sciences University, Namakkal, 637 002 India; 3 Department of Biotechnology, Annai College of Arts and Science, Kumbakonam, Tamil Nadu India; 4 Centre for Drug Discovery and Development, Sathyabama Institute of Science and Technology, Chennai, Tamilnadu 600119 India; 5 Department of Zoology, College of Science, King Saud University, PO Box2455, Riyadh, 11451 Saudi Arabia; 6 State Key Laboratory of Biobased Materials and Green Paper Making, School of Food Science and Engineering, Qilu University of Technology, Shandong Academy of Sciences, Jinan, Shandong People’s Republic of China; 7 Stem Cell and Molecular Biology Lab, Department of Biotechnology, Bhupat and Jyoti Mehta School of Biosciences, Indian Institute of Technology Madras, Chennai, 600036 India; 8 Amity University, Mumbai, India; 9 US Department of Veterans Affairs, Loma Linda University, Loma Linda, CA 92354 USA; 10 Nanobiomedicine Lab, Department of Pharmacology, Saveetha Institute of Medical and Technical Sciences, Saveetha Dental College, Chennai, Tamil Nadu India; 11 Centre for Materials Engineering and Regenerative Medicine, Bharath Institute of Higher Education and Research (BIHER), Chennai, India; 12 Department of Chemistry and Biology, School of Natural and Applied Sciences, Mulungushi University, 80415 Kabwe, Zambia

**Keywords:** Drug delivery, Pharmacology

## Abstract

Recent breakthroughs in the field of nanoparticle-based therapeutic delivery methods have changed the standpoint of cancer therapy by effectively delaying the process of disease development. Nanoparticles have a unique capacity of good penetrating ability than other therapeutic leads used in traditional therapeutics, and also, they have the highest impact on disease management. In the current study isolongifolene-loaded Chitosan nanoparticles have been formulated, synthesized and then characterized by the use of Fourier Transform Infrared Spectroscopy, X-ray Diffraction, Scanning Electron Microscopy and Transmission Electron Microscopy. Further, the characterized chitosan nano formulation was evaluated for hemocompatibility, plasma stability, and in-vitro release. Isolongifolene-loaded chitosan nanoparticles were found to be compatible with plasma and also, they exhibited a constant release pattern. Hence, chitosan-loaded nanoparticles could be employed as an excellent adjuvant in cancer therapeutic, to combat the multi-drug resistance in solid tumors.

## Introduction

The route of drug delivery to the targeted population is meant for effective illness management and therapy. The main problem with the current conventional way of drug delivery system is that the medicine does not reach the target as they cannot pass the micro-capillaries. Another method is the use of liposomes as potential carriers has the fascinating benefit of protecting pharmaceuticals from degradation, promoting focused action, and reducing systemic toxicity. However, several drawbacks, including limited encapsulation efficiency, quick leakage of water-soluble drugs in the presence of blood components, and poor storage stability, have led us to seek a better alternative to liposome-based administration. So, as an alternate, nanoparticles can be used for delivery processes, which are particulate dispersions or solid particles with a size range of 1–100 nm that could be used as a matrix to help transport the therapeutic lead molecule either (via) dissolved, entrapped (or) attached mechanisms.

These nanoparticle-based therapeutics have the potential to revolutionize the treatment of cancer^1^. The nanoparticles could be either synthesized as nano-spheres or nano-capsules depending on the method of preparation^1–5^. Nano-capsules are systems in which the drug is incorporated within the system's core and protected by a unique polymer membrane. While the nanospheres are matrix systems that produce homogenous drug dispersions^6^. Currently, multidimensional nanotechnology platforms in the development or clinical stages are being investigated to generate more effective and safer treatments with lesser side effects.

Polymers are the most promising materials for making numerous molecular patterns and integrating them as distinct nanoparticle constructions for biological purposes, particularly cancer treatments. Intravenous injections have been reported by researchers. Biodegradable polymeric nanoparticles, particularly those coated with hydrophilic polymers such as poly ethylene glycol (PEG), are useful as drug delivery devices that target a specific organ^2,5^.

Polymeric nanoparticles have reportedly emerged as a viable alternative to the aforementioned liposome method, owing to their enhanced drug/protein stability and long-term drug release properties^7,8^. The size of polymeric nanoparticles has a significant impact on medication retention in the bloodstream. A previous study was published that used a sterically stabilized ligand nanoparticle formulation for the targeted administration of antisense oligodeoxynucleotides and small interfering RNA into lung cancer cells^9,10^. So, it is clearly evident that the carrier or the delivery system via a polymeric nanoparticle is highly achievable and target specific.

Chitosan is a polymer formed from partial deacetylation of chitin that is common in crustacean and insect shells. It is made up of repeating units of glucosamine and N-acetylglucosamine, and the quantities control the degree of polymer deacetylation. At neutral pH, chitosan is insoluble, whereas it is soluble and positively charged at acidic pH^11^. Chitosan comes in a variety of molecular weights. In comparison to their high-molecular-weight relatives, low-molecular-weight and low-deacetylating chitosans have greater solubility and faster breakdown. Chitosan has also been shown to have antimicrobial, antifungal, and wound-healing effects. A previous study using the chitosan for assessing the LD (lethal dose) showed an oral LD50 of over 16 g/kg body weight in mice proving it to be non-toxic and most significantly biodegradable^11^.

Sodium alginate is a polymer made up of alginic acid and D-mannuronic acid and L-guluronic acid residues. To create a chain, these units are joined together by − 1, 4, and − 1, 4 glycosidic bonds^12^.

Gelatin is a biopolymer with a fine biocompatibility and biodegradability making it an ideal choice for pharmaceutical and medical applications^13^. In a previous finding, a size of 100 nm gelatin particles after delivery were found to be preferentially clustered around the leaky tumor vasculature and failed to permeate the interstitial space's strong collagen matrix. Furthermore, the MMP-2 activity destroyed the gelatin core of the 100 nm particle, allowing smaller 10 nm particles to emerge from the surface. Because of their reduced size, these MMP-2 modified particles can penetrate deep into tumors^14^. Isolongifolene (ILF), a carbazole alkaloid isolated from the curry leaf plant—*M. koenigii*, an Indian herb have been proven to possess with a wide range of therapeutic characteristics^15^. Isolongifolene has a woody and amber incense odor and is used as a fragrance in cosmetics, perfumes, space sprays, detergents, deodorants, and fabrics. Isolongifolene is a commercially accessible sesquiterpene hydrocarbon with an Isolongifolene skeleton. In an in-vitro model of Parkinson’s disease (PD), it was found that ILF has neuroprotective effects against rotenone-induced pathological symptoms such as oxidative stress, mitochondrial malfunction, and apoptosis. In a rat model of rotenone-induced PD, ILF showed improved behavioral impairments and reduced oxidative damage. As a result, ILF appears to be a potential pharmacological candidate for the treatment^16^. Therefore, the need for a nano-sized particle delivery system of medicine is required for the successful delivery of medicinal lead into the host system.

In this study, we have formulated, synthesized and validated an isolongifolene-synthesized polymeric nano-formulation for more advantages and also established its compatibility with polymers like sodium alginate, chitosan and gelatin. The efficacy, stability, and cytotoxicity of the improved Nano formulations for cancer therapy were also investigated. As a result, our research focused on isolongifolene-loaded polymeric nanoparticles, which have great adjuvant properties and could be used to treat a variety of diseases.

## Materials and methods

### Materials

Isolongifolene, Chitosan, and Sodium tri poly-phosphate were procured from Sigma Aldrich (USA). All other chemicals used in this study were of analytical grande.

### Fabrication of isolongifolene loaded chitosan nanoparticles

Isolongifolene-loaded Chitosan Nanoparticles (ICN) were fabricated by ionic cross-linking of chitosan with sodium tripolyphosphate (TPP) anions using a reported procedure^17^. In brief, 2 mg/mL of chitosan was dissolved in 0.25%v/v acetic acid under constant stirring conditions at 10 °C for 12 h. About 0.75% w/v of TPP (aqueous solution) was added (2:1 ratio) into chitosan solution containing 100 μg/mL of isolongifolene (dissolved in methanol), with continuous stirring for 6 h at 4 °C. The resultant dispersion was centrifuged at 13,000 × g for 20 min at 4 °C. The supernatant was removed and ICN was carefully collected and stored at − 55 °C.

### FT-IR analysis

The ICN was characterized using FTIR spectroscopy (Perkin Elmer, Spectrum-RX1, USA) to analyze the chemical interactions between the isolongifolene and chitosan polymer. The scanning range for FTIR wasbetween 4000 and 400 cm^−1^.

### X-Ray diffraction study

An X-ray diffraction study was performed for both chitosan and ICN to confirm the amorphous nature of ICN. This experiment was performed by exposing the samples to Cu-Kα1 radiation of 40 kV and 30 mA. The scanning rate was5°/min over a range of 4–90°with an interval of 0.1°.

### Optimization of ICN

Different ICN nano formulations were prepared by varying the ratios of chitosan for the fixed amount of isolongifolene. The different concentrations of chitosan (0.1, 0.2, 0.3, 0.4, and 0.5% w/v) were obtained by dissolving in acetic acid (0.25% v/v) under constant stirring (250 rpm) for overnight. About 20 mL of TPP of 0.75% (w/v) was added into chitosan solution (10 mL) containing 0.20 mg of isolongifolene and constantly stirred at 320 rpm for 6 h at 4 °C. Sonication (Sonitvibra cell, UC130, USA, Amplitude-20, Pulser-4 s) was performed for 10 min at 4 °C. Then, the dispersion was washed thrice (2200 × g) and re-dispersed in HPLC grade water (LiChrosolv). Further, the resultant solution was centrifuged at 13,000 × g for 20 min at 4 °C to obtain five different Isolongifolene polymer ratio nano formulations (ICN-K01 (1:0.5), ICN-K02 (1:1), ICN-K03 (1:1.5), ICN-K04 (1:2) and ICN-K05 (1:2.5). The resultant nanoparticles were freeze-dried (Delvac-lyo1550, INDIA) and stored at − 20 °C for further characterization.

### Process yield

Process yield was calculated by weighing the freeze-dried nano formulations i.e., ICN-K01 to ICN-K05 using the reported method^18^. The mean values of three replicates were expressed as process yield.

### Calculation of drug encapsulation efficiency (DEE) and drug loading efficiency (DLE)

The drug encapsulation efficiency (DEE) and drug loading efficiency (DLE) were calculated following the standard method reported earlier with some modifications^19^. Further, ICN was pelletized at 5800 × g and the obtained supernatant was quantified for isolongifolene by HPLC as described earlier. The percentages of DEE and DLE were calculated using the formula given below:-

$${\text{DEE}} = \frac{{{\text{Total}}\;{\text{amount}}\;{\text{of}}\;{\text{drug - drug}}\;{\text{in}}\;{\text{supernatant}}}}{{{\text{The}}\;{\text{total}}\;{\text{amount}}\;{\text{of}}\;{\text{drug}}}} \times 100$$

$${\text{DLE}} = \frac{{{\text{Total}}\;{\text{amount}}\;{\text{of}}\;{\text{drug - drug}}\;{\text{in}}\;{\text{supernatant}}}}{{{\text{Weight}}\;{\text{of}}\;{\text{recovered}}\;{\text{nanoparticles}}}} \times 100$$

### In vitro drug release study of isolongifolene

In vitro drug release was studied by plasma simulation^20^ and dialysis membrane method with slight modifications. The nanoformulations were redispersed in 10 mL of NaCl (0.9% w/v) with final Isolongifolene concentration of 20 μg/mL. 1 mL of the above-mentioned mixture was added to 10 mL of PBS (0.5 M, pH = 5.5) and 10 mL of plasma. The suspension was kept in an orbital shaker at 37 °C. One milliliter of the released solution was collected at different time intervals and replaced with a fresh solution. The harvested solutions were centrifuged and the supernatant was used to analyze isolongifolene content using the HPLC method. The release rate was calculated using the following equation.

$${\text{Isolongifolene}}\;\left( \% \right) = \frac{{{\text{Amount}}\;{\text{of}}\;{\text{Isolongifolene}}\;{\text{released}}\;{\text{at}}\;{\text{a}}\;{\text{time}}^{^{\prime\prime}} {\text{t}}^{^{\prime\prime}} }}{{{\text{The}}\;{\text{Amount}}\;{\text{of}}\;{\text{Isolongifolene}}\;{\text{loaded}}\;{\text{in}}\;{\text{the}}\;{\text{nanoformulation}}}} \times 100$$

In the dialysis membrane method, 2 mg of nanoformulations were redispersed in 10 mL of PBS (0.5 M, pH = 5.5), placed in a dialysis membrane (cut-off 10 kDa) and dialyzed against PBS (0.5 M pH = 5.5). 2 mL of released solution was collected at different time intervals, replaced with fresh solution and analyzed using HPLC. The release rate of Isolongifolenecan is derived from the calibration curve prepared using known concentrations.

### Size, shape, and zeta potential measurement

The size distribution and zeta potential of different nanoformulations (ICN-K01–ICN-K05) were measured by Zetasizer Nano ZSinstrument (Malvern, Mastersizer 2000, UK). The sample was dispersed in water (pH = 5.5) and the nanoparticles were counted in a 4.8 mm calibrated area with a count rate of 210.3 kcps (kilo counts per second) for 70 s. The average hydrodynamic diameter of different nanoformulations was calculated as mean values. The surface morphology of ICN was analyzed using Field Emission-Scanning Electron Microscopy (FE-SEM) (TESCAN, VEGA3 SBU, Czech). The average particle size and shape of ICN were further studied using High Resolution-Transmission Electron Microscope (HR-TEM) (Jeol, JEM2100, Japan).

### Stability of ICN in blood plasma

Fresh blood from Wistar rats was collected in heparinized tubes and the plasma was separated by centrifugation (1800 × g for 15 min at 4 °C). The 10 μg conc. of ICN-K04 was added into the separated plasma and further incubated at 37 °C for 30 min in 0.9% (w/v) of NaCl solution. 1 mL plasma solution was collected at regular time intervals and stored at − 20 °C until use. The isolongifolene content was analyzed by the HPLC method after centrifugation at 17,000 × g for 20 min. This study was carried out in compliance with the CPCSEA safety guidelines and following the ARRIVE Guidelines (https://arrivedguidelines.org) for the reporting of animal experiments. The study was approved by the Institutional Animal Ethics Committee (IAEC) of Periyar University Approval No: 1085/ac/07/PU/IAEC/Feb2012/04.

### Hemocompatibility study

The hemocompatibility of ICN was evaluated using the reported procedure^21^. The whole blood was collected from a Wistar rat and anticoagulated with sodium citrate (ratio of blood to anticoagulant taken was 9:1). Erythrocytes were isolated by centrifuging whole blood at 1000 × g for 10 min. The erythrocytes were washed thrice with saline before use. ICN-K04 was mixed with RBCs in different concentrations (2–24 μg/mL) and then incubated for 2 h at 37 °C and the supernatant was collected by centrifugation at 1500 × g for 5 min. Hemoglobin release was monitored spectrophotometrically (Systronics, 2203, INDIA) at 541 nm. The TritonX-100 (1% v/v) and 0.9% (w/v) NaCl were taken as positive and negative controls respectively. The percentage of hemolysis was calculated using the following formula:-

$${\text{Hemolysis}}\,\left( \% \right) = \left( {{\text{ODtest}} - {\text{ODneg}}{.}} \right)/\left( {{\text{ODpos}}{.} - {\text{ODneg}}{.}} \right) \times 100$$

where ODtest, ODneg., and ODpos are the absorbance values of the test sample, negative control, and positive control, respectively.

### Measurement of cell viability

Cell viability was analyzed using a conventional MTT reduction assay. Cells were treated with ICN and the viability was assessed based on the detection of mitochondrial dehydrogenase enzyme activity in viable cells^22^. Cells were cultured in a 96-multiwell plate. The 3 × 103 cells were seeded to each well. Initially, the cells in the medium were pre-incubated with or without ICN for 24 h. After 24 h, the cells were incubated with MTT (5 mg/mL) at 37 oC for 4 h. Following incubation, the medium was removed and the formed formazan crystals were dissolved with DMSO. The absorbance of the reduced product, formazan was measured at 570 nm using an ELISA plate reader (Bio-Rad, Hercules, CA, USA). The percentage of cell viability can be determined using the below formula:-

$${\text{Cell viability}} \left( \% \right) = \left( {\left[ {{\text{O.D. of control}} - {\text{O.D. of test compound}}} \right]/\left[ {{\text{O.D. of control}}} \right]} \right) \times 100$$

where O.D represents Optical Density.

### Apoptosis study

The A549 cell lines were procured from ATCC, USA and were used for apoptotic studies. The Apoptosis assay was performed using both acridine orange (AO) and ethidium bromide (EtBr) dyes. Acridine orange, a permeable dye stain all the cells, and ethidium bromide permeate into the cell only when the cell membranes disintegrate. EtBr intercalates with DNA forming an orange-red complex. Once the medium was removed from the plates after treatment, the cells were washed twice with phosphate-buffered saline (PBS) and stained with AO and EtBr dyes. The stained cells were incubated for 20 min at room temperature and washed with warm PBS to remove the excess dye. The cellular morphology was observed using a fluorescent microscope (λex/λem = 490 nm/530 nm) and the images were captured. The fluorescent intensity was recorded at 535 nm using Spectro-fluorimeter.

### Ethical approval

The present study follows institutional guidelines mandatory for human and animal treatment and complies with relevant legislation ethical approval from the institute for conducting the research. The study was conducted according to the ethical norms approved by Institutional Animal Ethical Committee of Periyar University Approval No: 1085/ac/07/PU/IAEC/Feb2012/04.

## Results

### FT-IR analysis

**Figure 1:**
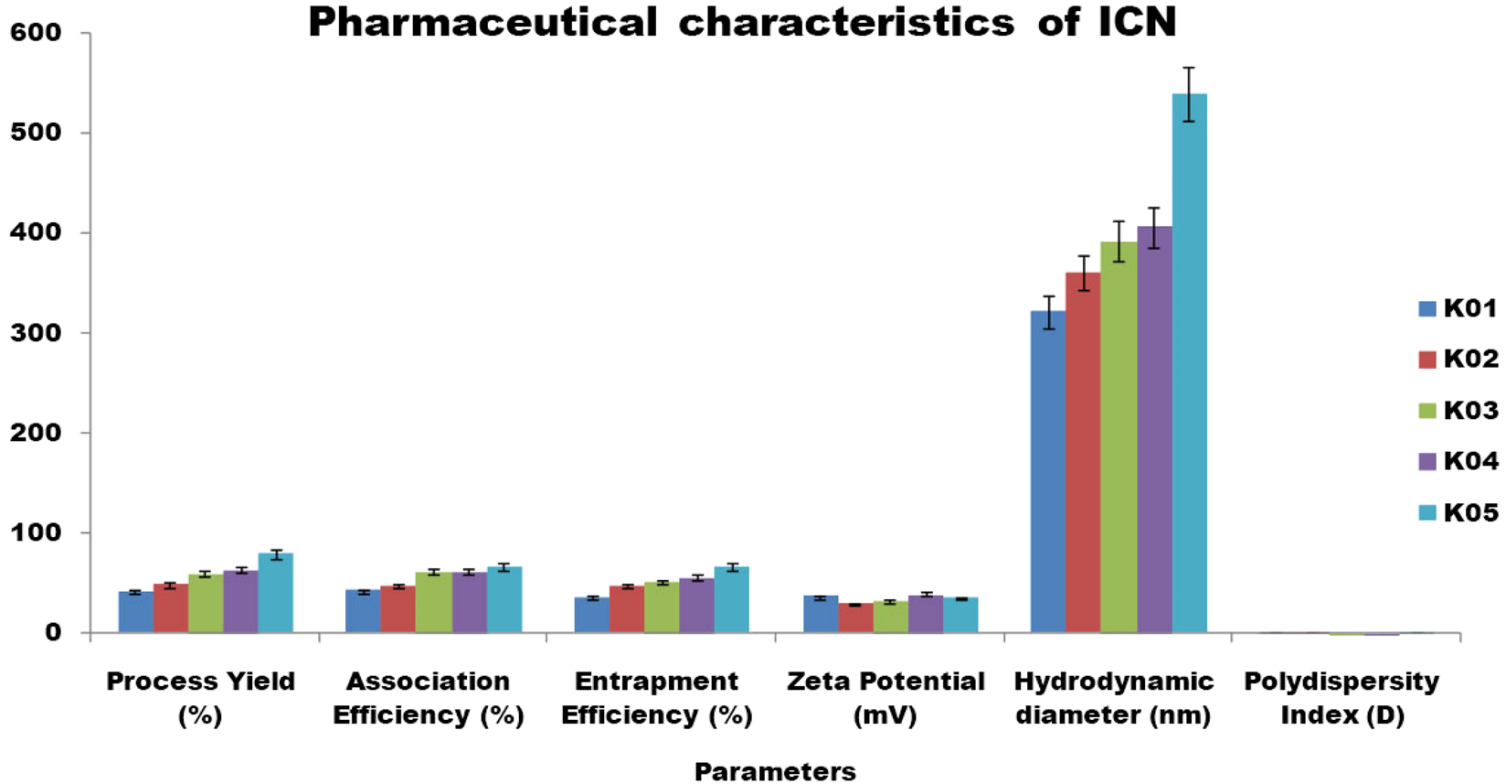
Statistical representation of pharmaceutical characteristics of ICN.

**Figure 2:**
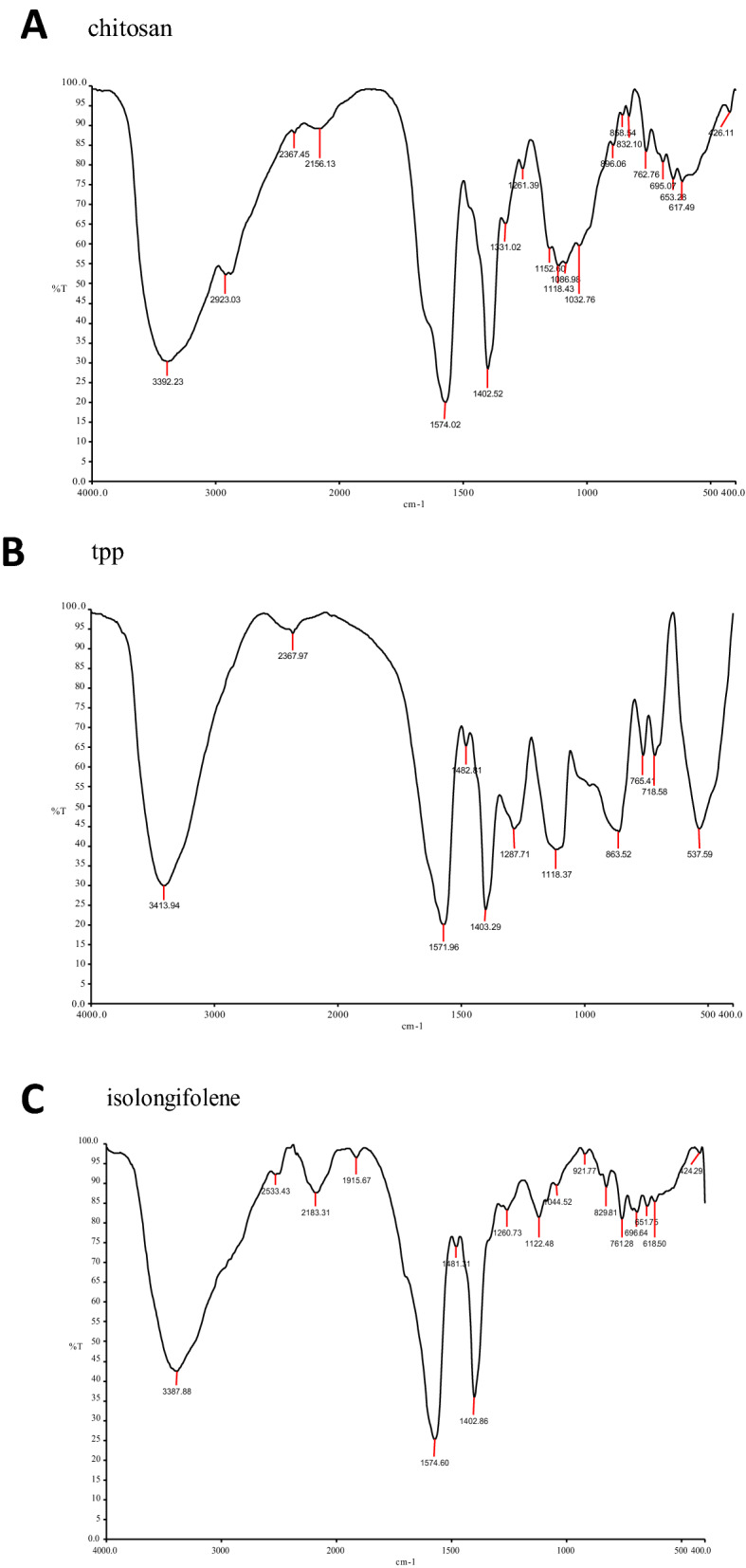

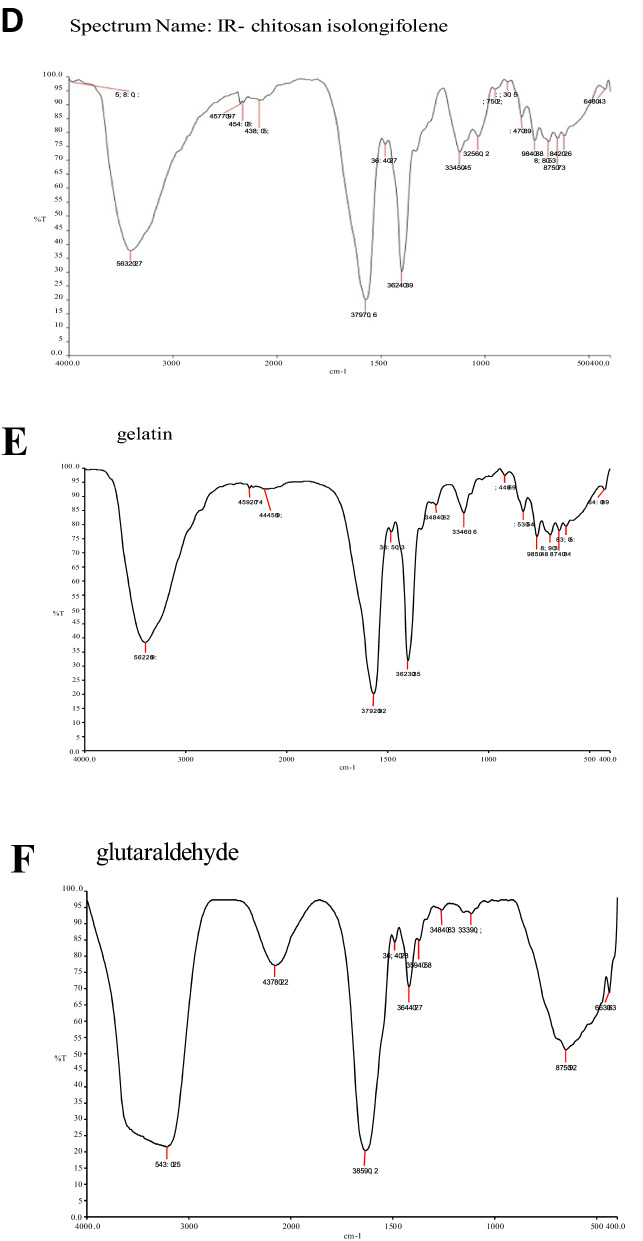

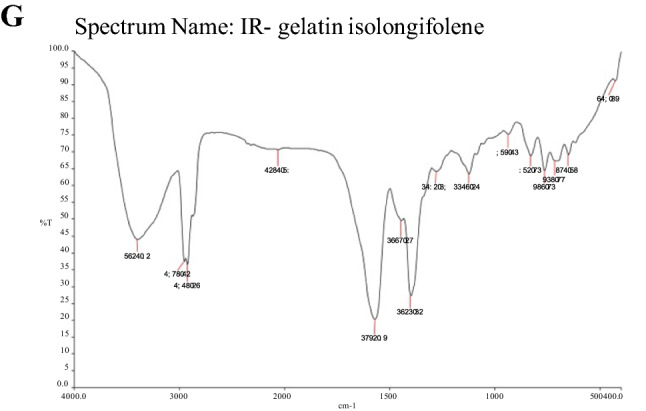
FT-IR spectra of Isolongifolene-Chitosan compatability. (**A**,**B**): FTIR spectrum of Chitosan, TPP, Isolongifolene: (**A**)—stretch in 3392 cm/1 (N–H) (**B**)—stretch in 1574 cm/1 (N–N). (**C**,**D**): FTIR spectrum of isolongifolene and cross linked isolngifolene. (**A**)—1482 cm/1; (**B**)—3410 cm/1 and 1482 cm/1 stretch were found. (**E**,**F**): FTIR spectrum of Gelatin and Glutaraldehyde. (**A**)—strech in 3400 cm^−1^, (**B**)—stretch in 1262 cm/1. (**G**): FTIR spectrum of Gelatin isolongifolene . (**A**)—Fails to possess isolongifolene Strech in 1482 cm/1.

The FT-IR spectrum of isolongifolene polymers such as sodium alginate, gelatin, and chitosan and their interaction with isolongifolene were shown in Fig. 1. The FTIR spectra of isolongifolene shows signature peaks at 1242 cm^−1^ (C–O stretch), 1407 cm^−1^ (C–C stretch), 1590 cm^−1^(C=O stretch), 2965 cm^−1^(C–H stretch), 3688 cm^−1^(O–H stretch). ISN spectrum showed significant peaks at 3218 cm^−1^(O–H vibration) and 1673 cm^−1^(C=C stretching). However, an ISN spectrum lacks an FT-IR band at 1242 cm^−1^corresponding to the C–O stretch of isolongifolene (Fig. 2).

### Optimization of isolongifolene to biopolymer ratio

To find the effective isolongifolene chitosan polymer ratio, different concentrations of chitosan were varied for a fixed amount of isolongifolene. The higher the amount of isolongifolene in polymer, higher the efficiency. Moreover, the increase in the Isolongifolene Chitosan ratio also increases the process yield as observed for the formulation ICN-K01 to ICN-K05. The formulation ICN-K05 which comprises of 1:2.5 ratio of isolongifolene chitosan polymer displayed a higher process yield (79.05 ± 4.60%). The association and entrapment efficiency of formulations increased with an increase in the isolongifolene chitosan ratio from 1:0.5 to 1:2.5. Figure 1 shows the statistical significance level of pharmaceutical characteristics of isolongifolene (ICN).

The hydrodynamic diameter of the different nanoformulations (ICN-K01 to ICN-K05 ranging from 322 to 538 nm was observed. Transmission electron microscopic (TEM) analysis revealed the particle size was found within the size range of 200–250 nm as shown in Fig. 4. Increased particle size seen with higher isolongifolene chitosan polymer ratio could be due to the increased swelling and subsequent polymer interaction. The polydispersity index (PDI) of the nanoformulations (ICN-K01 to ICN-K05) indicates the width of particle size distribution and is found to vary between 0.177 and 0.404. Amongst, the formulation K04 exhibited low PDI (0.195) when compared to other isolongifolene polymer ratios studied.

### In-vitro release determination

**Figure 3:**
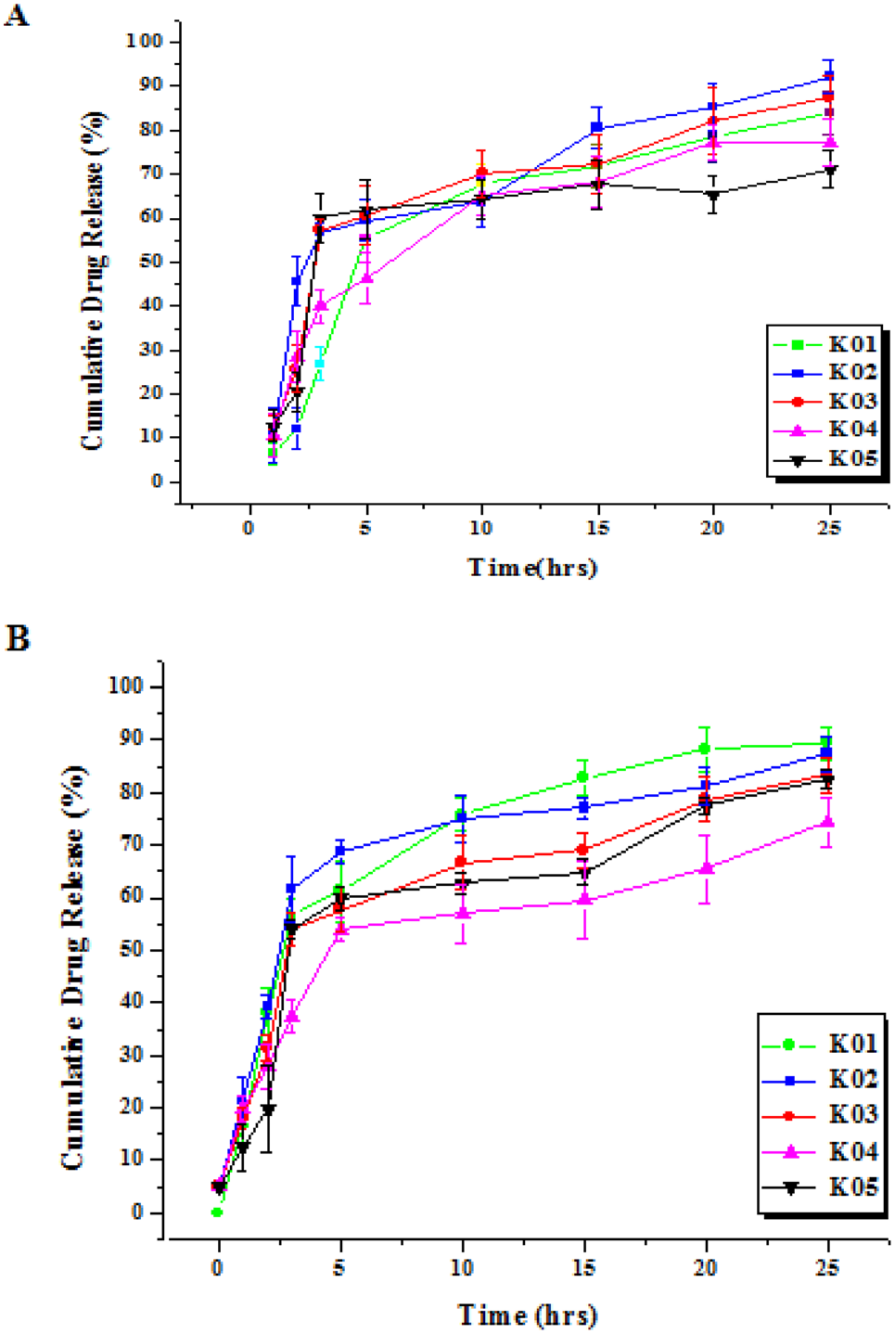
In-vitro release profiles of different nanoformulations estimated by (**A**) plasma simulation method, (**B**) Dialysis membrane method. Values are expressed as mean ± SE, n = 3.

The release pattern of nanoformulations ICN-K01 to ICN-K05 was analyzed by plasma simulation and dialysis method. There was no significant difference in the release pattern of nanoformulations studied using plasma simulation and dialysis membrane methods respectively (Fig. 3A,B).

The release profile of isolongifolene was directly proportional to the size of ICN. The release pattern of isolongifolene in 25 h was gradually decreased due to the increased size of ICN. About 50% release of isolongifolene was attained in 3–10 h.

### Characterization of ICN-K04

**Figure 4:**
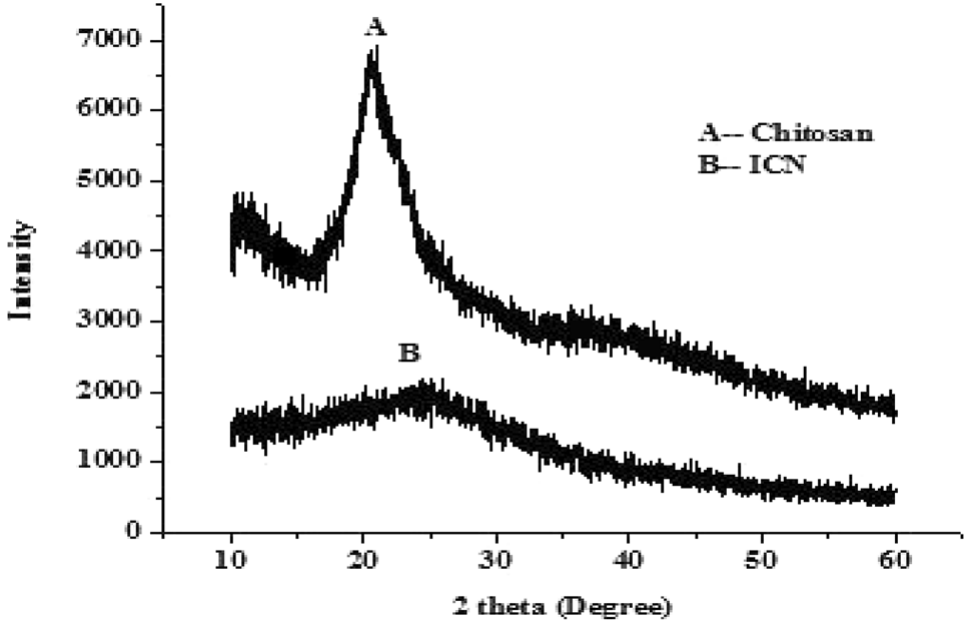
XRD spectrum of chitosan and Isolongifolene chitosan nanoparticle.

**Figure 5:**
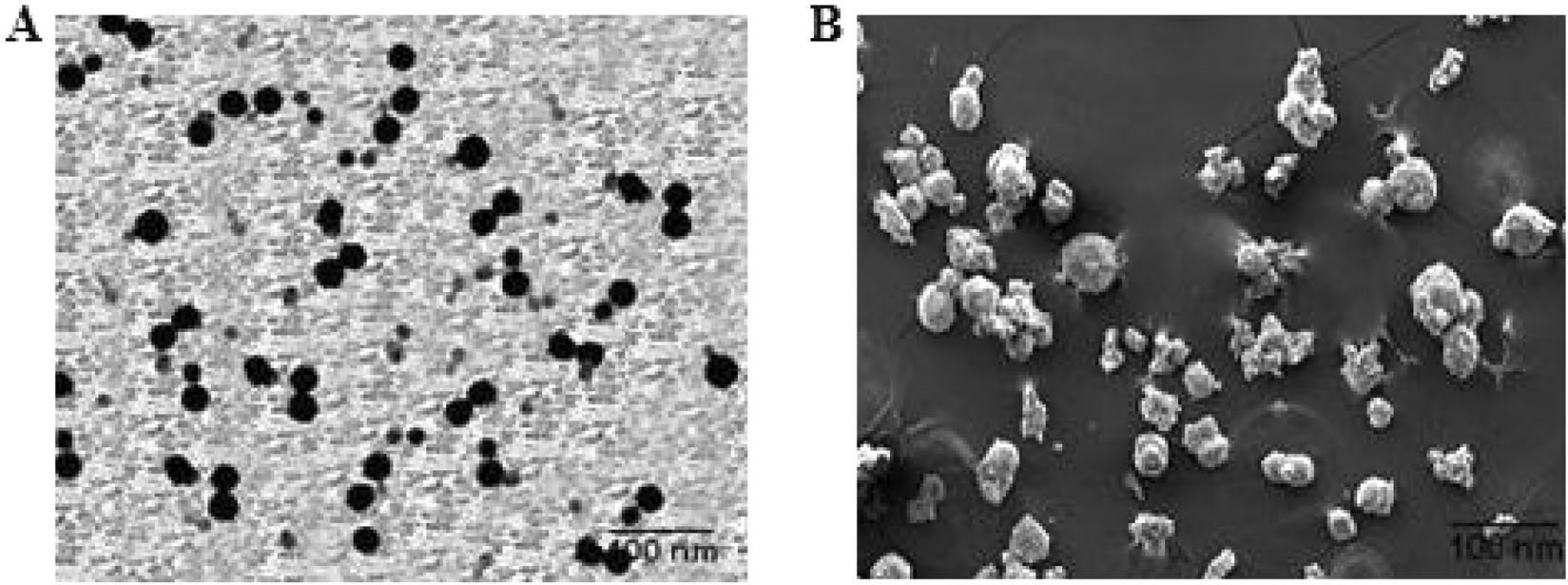
Electron Microscopic analysis of ICN-K04: (**A**) Transmission electron micrographs of ICN-K04shows particle size of 50–80 nm, (**B**) Scanning electron micrographs. Scale bar-100 nm.

The XRD spectrum of chitosan has shown a sharp peak at 25 °C which was found to be diminished in the case of ICN as shown in Fig. 4. The formation of Spherical shaped ICN-K04 nanoparticles was confirmed under transmission and scanning electron microscopic studies as shown in Fig. 5.

### Hemocompatibility of ICN-K04

**Figure 6:**
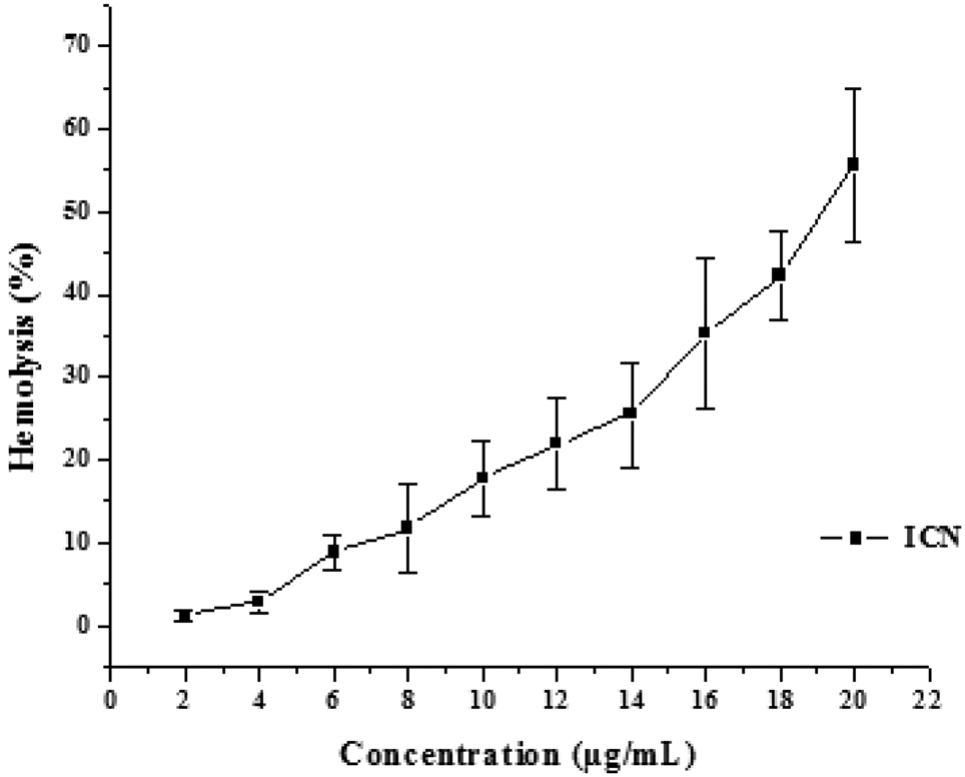
Hemocompatability of ICN-K04 at different concentration ranging from 2 to 20 μg/ml.

The hemocompatibility of ICN-K04 was studied by varying the concentration of ICN-K04 for a fixed amount of erythrocytes. It was observed that there was no significant damage occurred to erythrocytes till 14 μg/ml of ICN-K04. Above 14 μg/ml, the increase in nanoparticle concentration has shown to incur significant damage to erythrocytes and the phenomena were found to be concentration-dependent (Fig. 6).

### Genotoxicity of ICN-K04

**Figure 7:**
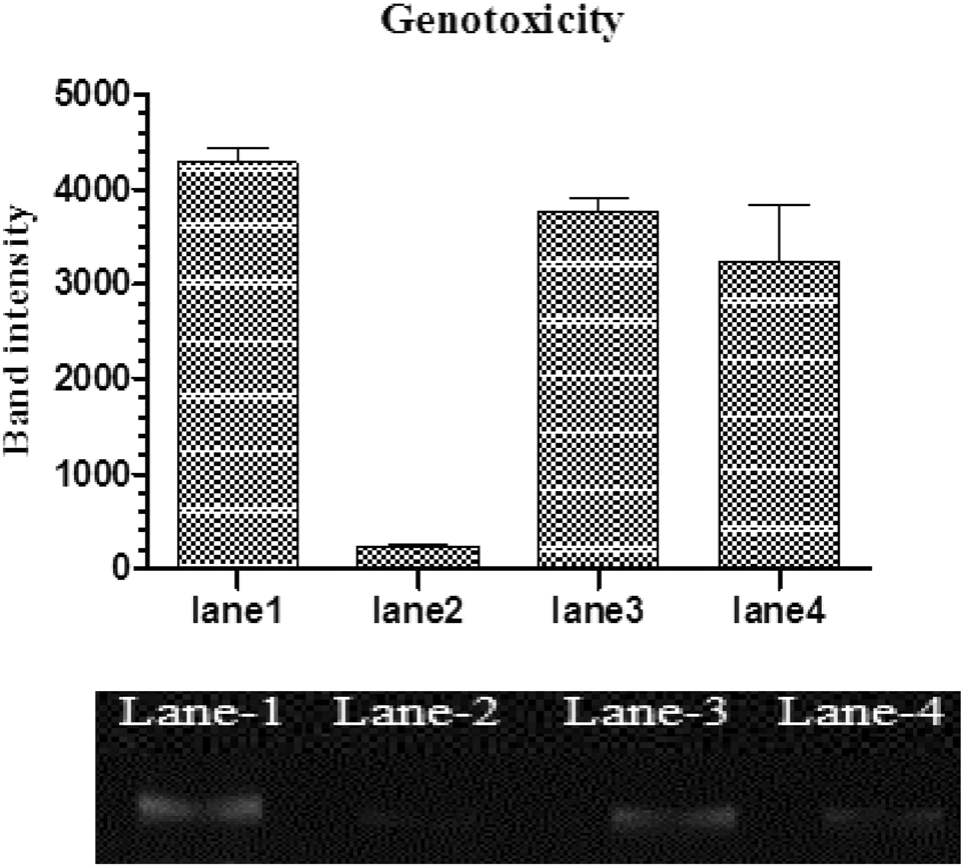
Genotoxicity of ICN-K04.Lane 1: pUC19 plasmid alone. Lane 2: plasmid treated with Fenton’s reagent. Lane 3: Plasmid + ICN-K04 (5 µg) and Lane 4: Plasmid + ICN-K04 (10 µg) respectively.

Genotoxicity was investigated by a plasmid nicking assay. Results showed that the band intensity corresponding to pUC19 plasmid alone in lane 1 was kept as control (100% intensity). However, the plasmid subjected to Fenton's reagent showed moderate DNA fragmentation with the lowest band intensity of 25%. The addition of 5 and 10 µg ICN-K04 did not incur significant damage to the plasmid with band intensities of 85% and 80% respectively (Fig. 7).

### Plasma stability of ICN-K04

**Figure 8:**
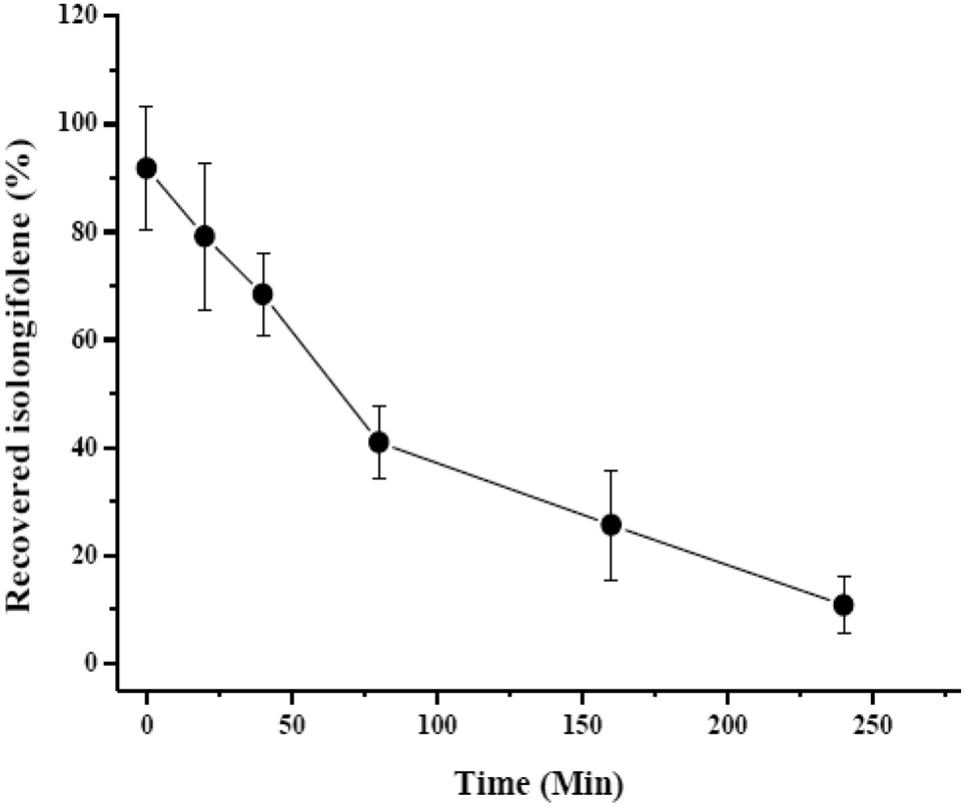
Plasma stability studies ofICN-K04 at different time intervals.

Plasma stability studies revealed that the formulation ICN-K04 was moderately stable up to 50 min and the stability gradually decreases further, as shown in Fig. 8.

### Effect of ICN-K04 on A549 cell viability

**Figure 9:**
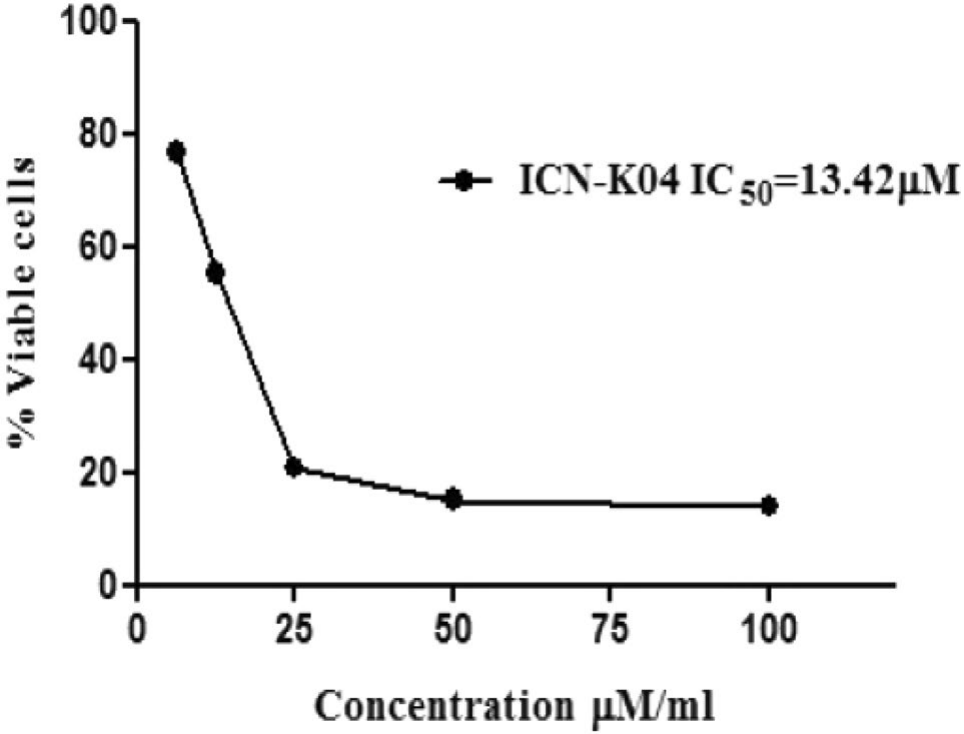
Cytotoxic potential of ICN-K04 (6.25, 12.5, 25, 50, 100 μM) measured using MTT assay in A549 cells for 24 h. The half-maximal inhibitory concentration (IC50) is obtained at 13.42 μM and error bars represent the mean ± SEM of three independent experiments.

*Invitro* cytotoxicity of the nanoformulation ICN-K04 of varying concentrations (6.25, 12.5,25,50,100 μM) was investigated using an MTT assay on the A549 cell line for 24 h as shown in Fig. 9. Live cells reduced the MTT and the resultant formazan is directly proportional to the cell viability. Results showed that the viability of cancer cells was inhibited in a dose-dependent manner with a 50% inhibitory concentration (IC50) of 13.42 μM. The addition of 25 μM of ICN-K04 decreased the % cell viability to 23.7%. For further studies, the half-maximal concentration (IC50) of ICN-K04 was used unless otherwise mentioned.

### Effect of ICN-K04 on apoptosis

**Figure 10:**
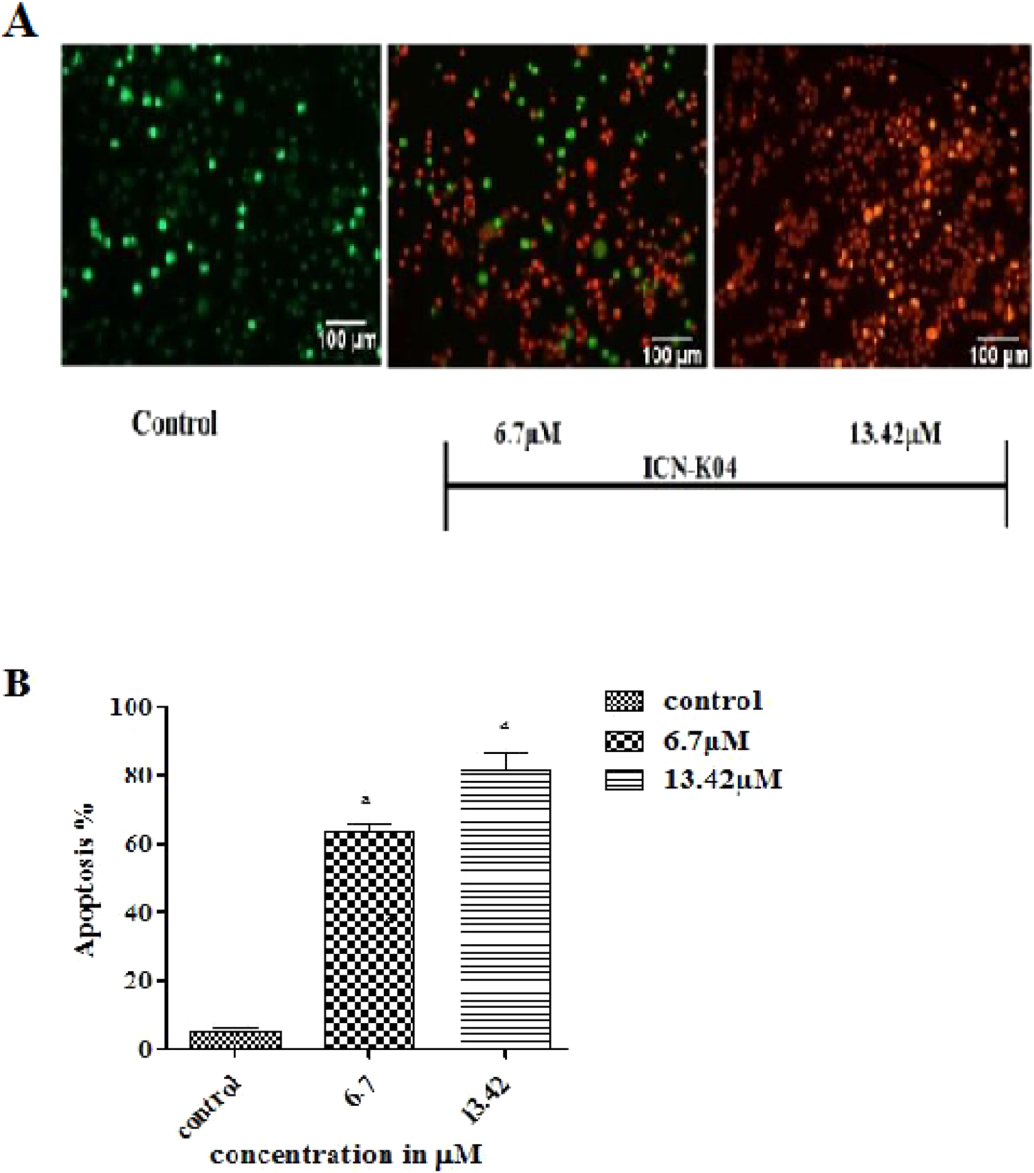
Apoptotic effect of ICN-K04. (**A**) Photomicrograph showing the effect of ICN-K04 induced apoptotic morphological changes in A549 cells. (**B**) Changes in fluorescent intensity after treatment with ICN-K04 in A549 cells as compared to control cells after 24 h. Values are expressed as Mean ± SEM. *P* < *0.05*.

Morphological alterations in A549 cancer cells such as viability, nuclear morphology, and chromatin condensation were evaluated by AO/EB staining technique. This method differentiates the viable cells from dead cells by monitoring the bright green nuclei and orange to red nuclei, depending on the loss of membrane integrity. Fluorescent microscopic studies of AO/EB stained A549 cells after treatment with ICN-K04 were shown in Fig. 10. Untreated cancer cells (control) displayed green fluorescent nuclei with normal cellular morphology. Whereas, cancerous cells treated with 6.7 and 13.42 μM ICN-K04 showed a significantly (*P* < *0.05*) increased percentage of apoptotic cells.

## Discussion

Hitherto, the major clinical challenge in treating the cancer is the relapse prevalence, which occurs due to the failure of the primary treatment regimen in targeting the cancerous cells. Therefore, drugs having the potential of huge accessibility and specifically delivered to the target site hold a great promise in the management of cancer. Nanoparticle-based drug delivery system has gained huge attention due to its versatile approach to accessing the cancerous site and another inflammatory milieu. Thus, the synthesized nanoparticles have a unique potential to overcome the poor penetrating ability and also exert their maximum efficacy to control the proliferation of the disease.

The current study investigated the compatibility of Isolongifolene nanoformulation with different polymers. Preliminary studies showed that the chitosan-based Isolongifolene polymeric nanoformulation could act as an excellent adjuvant in therapeutics, mainly treating multi-drug resistance in solid tumors.

In the FT-IR spectroscopic study, the peaks revealed the surface chemistry of the presence of functional groups other than the innate molecules as confirmed by the signature FT-bands with different wavenumbers corresponding to different significant changes obtained in shape and position of the absorbance bands^23^. Among the different ratios investigated, the 1:2.5 ratio of isolongifolene and polymer ratio yields a higher process yield, which could be due to greater carbon efficiency. In an experimental condition reduction of pH leads to greater association and loading efficiency^24^.

Zeta potential is an important physicochemical property that reflects the physical stability and mucoadhesive properties of nanoparticles^25^. In principle, zeta potential values in the range of <− 30 mV and > + 30 mV are considered stable regimes. In the present study, the ICN-K04 formulation possesses a zeta potential of +39 mV among others, which demonstrates its good interaction stability. Polydispersity Index (or) PDI is the measure of the particle size distribution with values ranging from 0 to 1. In this study, the PDI values close to 0 (zero) presented a homogeneous dispersion and those greater than 0.5 showed high heterogeneit^26^. The prepared nano formulations (ICN-K01 to ICN-K05) showed PDI values in the range of 0.1 to 0.4. Especially, ICN-K04 possesses a PDI of 0.1 which indicates the presence of monodispersed (homogenous) particles.

Moreover, the nanoformulation ICN-K04 showed increased association efficiency which was inversely proportional to the isolongifolene release. Subsequently, in vitro release of isolongifolene monitored by plasma simulation and membrane dialysis method showed the lack of burst effect and also confirmed that the interactions between isolongifolene and the nanoparticles were weak. ICN formulation was found to be optimum based on zeta potential, PDI value, and in vitro release methods, which demonstrate that ICN-K04 possesses good stability and homogenous dispersity. Further, the ICN-K04 formulations were used for the anti-carcinogenic study.

The ultra-structural image of the formulation revealed the presence of nearly shaped solid particles after the incorporation of isolongifolene. The formation of intermolecular hydrogen bonding did not show any significant shearing effect on the surface of ICN-K04. The biocompatibility of nano formulations used as drug delivery systems is a crucial parameter. The integrity of the hemoglobin structure might function as a key factor to determine the potential biocompatibility of nanoparticle. A few other mechanisms have been proposed whereby hemolysis can contribute to thrombosis in addition to changing the hematocrit and hemorheology, such as the release of erythrocyte-derived macrovesicles, activation of the complement cascade, and the release of free haemoglobin and heme into circulation, which sequesters nitric oxide. Hemolysis is a crucial factor in the hemocompatibility testing of biomaterials and can have a big impact on how well they work in the clinic. A number of NPs, including amorphous silica, tricalcium phosphate, hydroxyapatite, and particularly silver (Ag) NPs, have been discovered to significantly cause hemolysis, endangering their use in biomedical applications.

Most NPs have hemolytic activity; however, it depends on concentration, structure, size, and shape. For instance, the amount of reactive silanol groups exposed on the surface of silica NP is directly related to the size and geometry of the NP. Surface charge, shape, porosity, and surface functionalization with certain polymers or functional groups are the most important surface characteristics that determine the hemocompatibility of NPs.^27^. Upon administration, injected particles will most likely interact with red blood cells. Electrostatic interactions between the red blood cells and the nanoparticles can cause perturbation of the membrane, thereby causing hemolysis^28^. In the present experimental study ICN-K04 showed and induced hemolysis at higher concentrations, which could be due to the electrostatic attractive forces between chitosan and erythrocytes and subsequently lead to thrombus formation. Nanoparticle movements were faster as compared to macromolecules. It was also expected that the nano formulations could reach the tissue compartment within 50 min.

In cancer therapy, tumor growth can be suppressed by activating the apoptotic machinery in the cell^29^. Many malignant cells, however, are unable to regulate the genes that control apoptosis, rendering them resistant to the induction of apoptosis by a variety of stimuli, including intracellular and extracellular signals chemotherapeutic drugs, and radiotherapy^30^.

A previous study has investigated that the SH-SY5Y cells exposed to rotenone caused about 50% cell death, whereas isolongifolene pre-treated cells dose-dependently regulated the toxic effects of rotenone by which it exhibits neuroprotective effect against apoptosis^31^. In our study, we investigated the cytotoxicity of nanoformulation ICN-K04 at different A549 cell lines for 24 h. The result exhibited that the viability of cancer cells was inhibited by 50% in a dose-dependent manner, and also we found that 50% inhibitory concentration (IC50) of ICN-K04 at 13.42 μM among all other concentrations respectively.

## Conclusion

Isolongifolene loaded chitosan nanoparticles were successfully prepared and characterized for parameters like association efficiency, loading efficiency, zeta potential, entrapment efficiency, and polydispersity index (PdI). Among the nano formulations (ICN-K01to ICN-K05) prepared, the ICN-K04 formulation was found to be optimum. The X-ray diffraction spectrum of ICN-K04 formulation confirms the poor crystalline nature of the nanoparticles. The spherical morphology of ICN-K04 nanoparticles was confirmed by scanning and transmission electron microscopy. More importantly, ICN-K04 nanoparticles have low hemolytic activity and also lack genotoxicity. ICN-K04 nanoparticles show intrinsic plasma stability of more than 50 min. As a prerequisite for a nanoparticle-based drug delivery system, it is imperative to understand the size, morphology, as well as surface charge of nanoparticles which can deliver the loaded drug effectively and specifically to its targeted site. From the results it can be corroborated that the ICN-K04 nanoparticles show promising data in all the physiochemical criteria, so that they can be used as a delivery system for the drug to show conspicuous effects to the targeted site. Further, validation studies of this drug delivery approach using the nanoparticles system are underway.
